# Editorial: Potential of Extracellular Matrix Molecules in Pharmaceutical Development

**DOI:** 10.3389/fphar.2020.636026

**Published:** 2021-01-14

**Authors:** Zhong Zheng, Feng Chen, Xinli Zhang

**Affiliations:** ^1^ Department of Surgery, David Geffen School of Medicine, University of California, Los Angeles, Los Angeles, CA, United States; ^2^ School of Dentistry, University of California, Los Angeles, Los Angeles, CA, United States; ^3^ Central Laboratory, Peking University School and Hospital of Stomatology, National Clinical Research Center for Oral Diseases, National Engineering Laboratory for Digital and Material Technology of Stomatology, Beijing Key Laboratory of Digital Stomatology, Beijing, China

**Keywords:** extracellular matrix, pharmaceutical, small leucine rich proteoglycan, matricellular protein, scaffold

The extracellular matrix (ECM) is a complex cell-secreted network composed of a diverse array of fibrous proteins, proteoglycans, and proteolytic enzymes. Comprising approximately one-third of the human body, the ECM was initially considered an inactive, space-filling scaffold that solely provides structural support for cell growth. However, dysfunctions in the ECM have been associated with various human diseases. In this special issue of *Frontiers in Pharmacology*, Ahmad et al. contributed a minireview that manifests the current understanding of the major ECM components in muscle-associated conditions and their therapeutic potentials.

Meanwhile, the influence of some therapeutics on ECM is also broadly observed. For example, Tang et al. revealed that Anisodamine (ANI; also known as 7β-hydroxyhyoscyamine), a naturally occurring atropine derivative, not only effectively inhibited the apoptosis, senescence, and ECM degradation in the nucleus pulposus of intervertebral disc degeneration (IVDD) rats but also significantly promoted the synthesis of aggrecan and type II collagen. Considering the loss of ECM, particularly aggrecan and type II collagen, is a typical feature of IVDD, this study may provide intuitiveness for ANI’s protective effects on cartilage, in addition to its known anti-inflammatory, analgesic, antipyretic, and platelet-inhibitory actions. In this special issue, Yang et al. also reported that long noncoding RNA (lncRNA) growth arrest specific transcript 5 (GAS5) bolsters the expression of growth arrest differentiation factor 5 (GDF5)—which plays an essential role in articular cartilage maintenance by inducing ECM and α5 integrin expression. Interestingly, they also found that GAS5 serves as an osteogenic regulator of periodontal ligament stem cells *via* GDF5 and p38/JNK signaling pathway, suggesting the critical function of GAS5 and GDF5 on ECM homeostasis in both bone and cartilage tissues.

Moreover, accumulating evidence currently demonstrates that ECM is a dynamic structure that directly interacts with the living cells. For instance, during the force-induced orthodontic tooth movement (OTM), a process of bone and periodontal ligament (PDL) remodeling, disorganized and compressed ECM architecture promotes osteoclastic bone resorption on the pressure side. At the same time, the stretching ECM structure stimulates osteoblastic bone formation. In this special issue, Li et al. accurately analyzed the ECM fiber remodeling and osteoclast recruitment corresponding to stress distribution. Hence, ECM has the capacity to modulate cell function and behavior. Undoubtedly, a comprehensive understanding of the mechanisms underlying these ECM-related disease pathologies and therapeutical benefits would help develop novel treatment strategies.

Echo to these observations, some regenerative medicines targeting ECM components have been being developed, specifically since the new century. One exciting application of ECM-based-therapeutics is the use of decellularized ECM. By removing the cellular components, a decellularized ECM acts as a water-insoluble matrix and retains the physiological ECM properties that mimic the native microenvironment and support tissue regeneration. The intact biocompatibility, biodegradability, and bioinductivity of decellularized ECMs make them broadly applicable to various kinds of tissue regeneration. This strategy is particularly successful in bone repair applications. Thus, Lin et al. provided an overview of the function of multiple types of bone ECM and the applications of both ECM-modified and decellularized ECM scaffolds in bone repair and regeneration.

Furthermore, matricellular molecules that do not simply function as structural blocks are also critical components of the ECM. Compared with fiber-forming ECM elements, such as collagen, matricellular molecules may be more applicable in pharmaceutical development. Indeed, significant efforts have been devoted to developing matricellular protein-based pharmaceuticals, especially cancer therapeutics and fibrotic and inflammatory diseases. Particularly, proteoglycans have emerged as biomacromolecules with critical ECM remodeling, homeostasis, and signaling roles in the past 2 decades. Here, Walimbe and Panitch contributed a review on the recent preclinical efforts that open new avenues for developing new and exciting treatments with proteoglycan cores and mimetics in a broad range of regenerative medicine. Pang et al. conducted a review with a particular interest in the diverse functions of small leucine-rich proteoglycans (SLRPs), a group of ECM that exist in a wide range of connecting tissues, in skin wound healing. In this review, the most recent knowledge of SLRPs’ anti-inflammatory, pro-angiogenic, pro-migratory, and pro-contractility, and signal transduction orchestrating effects, as well as their spatial-temporal expression in the skin, has been comprehensively summarized to pave the path for a new generation of pharmaceuticals discovery for patients suffering from skin wounds and their sequelae. In addition, by delineating the spatial-temporal distribution of fibromodulin (FMOD), an SLRP member, during cartilage and development, and FMOD’s structural alteration in aging and arthritis progression, Li et al. suggest that FMOD holds the high potential as a new target of osteoarthritis management, pointing a novel direction of arthritis pharmaceutical development.

In summary, this special issue aims to give an overview of the most exciting progress in ECM-based strategies for pharmaceutic development, although it is inevitably incomplete in covering all aspects of ECM studies. Many promising projects are in advanced experimental stages and/or preliminary clinical trials. Given the exciting developments in the field, we believe that this special issue will provide specific insights into the establishment of novel ECM-based strategies that can pave the path for these emerging ECM-relevant therapies to improve human health.

## Author Contributions

ZZ conducted the initial draft and FC and XZ revised the manuscript.

## Conflict of Interest

The authors declare that the research was conducted in the absence of any commercial or financial relationships that could be construed as a potential conflict of interest.

